# Advancing Personalized Medicine in the Treatment of Locally Advanced Rectal Cancer

**DOI:** 10.3390/jcm13092562

**Published:** 2024-04-26

**Authors:** Francesco Giulio Sullo, Alessandro Passardi, Chiara Gallio, Chiara Molinari, Giorgia Marisi, Eleonora Pozzi, Leonardo Solaini, Alessandro Bittoni

**Affiliations:** 1 Department of Medical Oncology, IRCCS Istituto Romagnolo per lo Studio dei Tumori (IRST) “Dino Amadori”, via P. Maroncelli 40, 47014 Meldola, Italy; francesco.sullo@irst.emr.it (F.G.S.); chiara.gallio@irst.emr.it (C.G.); alessandro.bittoni@irst.emr.it (A.B.); 2 Biosciences Laboratory, IRCCS Istituto Romagnolo per lo Studio dei Tumori (IRST) “Dino Amadori”, via P. Maroncelli 40, 47014 Meldola, Italy; chiara.molinari@irst.emr.it (C.M.); giorgia.marisi@irst.emr.it (G.M.); 3 Department of Medical and Surgical Science, University of Bologna, 47121 Forlì, Italyleonardo.solaini2@unibo.it (L.S.)

**Keywords:** rectal cancer, total neoadjuvant treatment, immune checkpoint inhibitors, nonoperative management, ctDNA

## Abstract

Rectal cancer presents a significant burden globally, often requiring multimodal therapy for locally advanced cases. Long-course chemoradiotherapy (LCRT) and short-course radiotherapy (SCRT) followed by surgery have been conventional neoadjuvant approaches. Recent trials favor LCRT due to improved local control. However, distant tumor recurrence remains a concern, prompting the exploration of total neoadjuvant therapy (TNT) as a comprehensive treatment strategy. Immune checkpoint inhibitors (ICIs) show promise, particularly in mismatch repair-deficient (dMMR) or microsatellite instability-high (MSI-H) tumors, potentially revolutionizing neoadjuvant regimens. Nonoperative management (NOM) represents a viable alternative post-neoadjuvant therapy for selected patients achieving complete clinical response (cCR). Additionally, monitoring minimal residual disease (MRD) using circulating tumor DNA (ctDNA) emerges as a non-invasive method for the assessment of treatment response. This review synthesizes current evidence on TNT, ICIs, NOM, and ctDNA, elucidating their implications for rectal cancer management and highlighting avenues for future research and clinical application.

## 1. Introduction

Colorectal cancer is the third most frequently diagnosed cancer globally, with rectal cancer accounting for approximately 31% of cases. Among patients with rectal cancer, 5 to 10% present with locally advanced disease (i.e., stage II-III) at the time of diagnosis. The standard approach for managing such cases involves multimodal therapy, combining radiotherapy, chemotherapy, and surgery [1,2,3]. For years, long-course chemoradiotherapy (LCRT) and short-course radiotherapy (SCRT), followed by total mesorectal excision (TME) surgery, have emerged as the mainstay neoadjuvant treatments for locally advanced rectal cancer (LARC) [4,5].

Evidence from the CAO/ARO/AIO-94 trial has established the superiority of preoperative LCRT over postoperative chemoradiotherapy in terms of local control, highlighting a 5-year cumulative incidence of local recurrence (LR) of 6% in the preoperative versus 13% in the postoperative arm [6]. Similarly, several studies have affirmed the efficacy of SCRT in reducing the risk of LR [7,8,9,10,11]. Despite randomized studies indicating comparable survival, local control, and late toxicity outcomes between LCRT and SCRT, the latter is associated with a clear reduction in complete pathological responses (pCRs), along with an increase in circumferential margin infiltration and LR. Therefore, while acknowledging the advantages of SCRT in terms of economic health and quality of life, the preference for LCRT prevails in cases where achieving a pCR is of utmost importance, as recommended by both clinical practice and international guidelines [12,13].

Despite these recommendations, a substantial proportion of patients (25% to 30% at 5 years) experience distant tumor recurrence. Given the debatable role of adjuvant chemotherapy [14,15,16], a compelling alternative has emerged—administering all systemic treatment before surgery. The introduction of total neoadjuvant therapy (TNT), involving upfront chemotherapy followed or preceded by either LCRT or SCRT, has ushered in a recent paradigm shift in managing LARC [17]. Additionally, the incorporation of immune checkpoint inhibitors (ICIs) in the neoadjuvant treatment for LARC has emerged as a promising avenue in recent oncological research, especially in mismatch repair-deficient (dMMR) or microsatellite instability-high (MSI-H) tumors [18].

Nonoperative management (NOM) is a viable option for selected LARC patients achieving a complete clinical response (cCR) post-neoadjuvant treatment. It involves active surveillance over immediate surgery, ensuring close monitoring for regrowth. 

Monitoring minimal residual disease (MRD) in LARC is crucial for assessing treatment efficacy and predicting the risk of recurrence. In this context, circulating tumor DNA (ctDNA) has garnered increasing interest as a potential biomarker for MRD. The presence and quantity of ctDNA may reflect residual tumor burden following neoadjuvant and surgical treatments. Therefore, ctDNA analysis holds promise as a non-invasive strategy for monitoring MRD in LARC, enabling more personalized and timely management of patients post-treatment [19].

**Figure 1:**
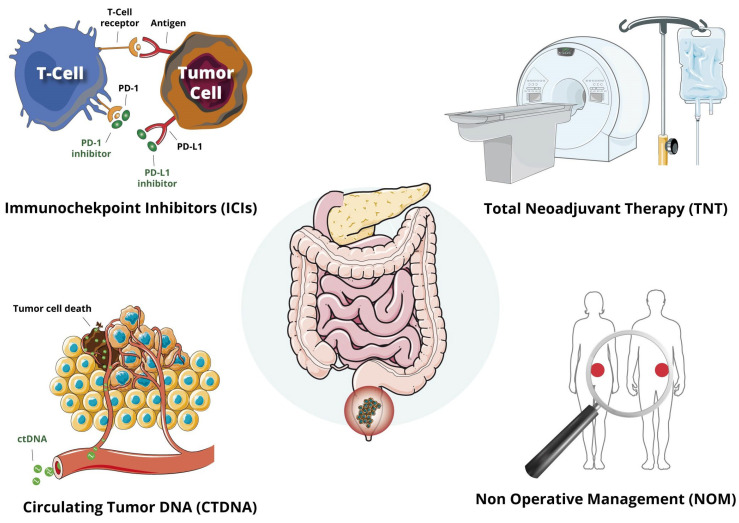
Advances in the multidisciplinary management of locally advanced rectal cancer.

This review aims to delve into the latest evidence regarding the utilization of TNT, ICIs, ctDNA, and NOM strategies in LARC. Through a comprehensive analysis of these topics, we aim to provide a thorough understanding of the new frontiers in LARC management, with particular emphasis on the potential clinical and therapeutic implications of these innovations (Figure 1).

## 2. Total Neoadjuvant Treatment (TNT)

### 2.1. Results of Phase III Trials

The introduction of LCRT in the management of LARC, followed by rectal resection with TME, has significantly reduced LR without a corresponding improvement in the rate of distant metastases, which remains at about 25–30%. Due to poor tolerance to adjuvant chemotherapy in this setting, different strategies of TNT have been explored in recent years through clinical trials. TNT is an alternative multimodal strategy aimed at intensifying preoperative treatment by delivering both radiotherapy and chemotherapy before surgery. This approach offers several theoretical advantages, including early treatment of micrometastatic disease and better compliance with chemotherapy compared to adjuvant treatment, albeit with the potential risk of overtreatment in some patients. In particular, two distinct approaches have emerged from several phase II and III trials: induction chemotherapy followed by LCRT, and LCRT or SCRT followed by consolidation chemotherapy. Recently, the results of three phase III trials, namely PRODIGE-23, RAPIDO, and STELLAR, comparing different TNT strategies to standard LCRT have been published (Table 1).

The PRODIGE-23 trial evaluated an induction TNT strategy, including chemotherapy with the mFOLFIRINOX regimen (5-fluorouracil, irinotecan, and oxaliplatin) for 3 months, followed by LCRT, surgery, and 3 months of adjuvant mFOLFOX (5-fluorouracil and oxaliplatin) or capecitabine, compared to standard LCRT and adjuvant treatment for 6 months. The study randomized 461 patients with LARC (cT3/cT4). The primary endpoint of the trial was disease-free survival (DFS), while secondary endpoints were toxicity, pCR, metastasis-free survival (MFS), overall survival (OS), and quality of life [20]. The study met its primary endpoint with an improved 3-year DFS in patients treated with TNT compared to standard treatment (76% vs. 69%, *p* = 0.034). The experimental arm also showed an increase in 3-year MFS (HR 0.69, 95% CI 0.54–0.90; *p* = 0.0048) and an increased rate of pCR (27.8% for TNT vs. 12.1% for standard treatment, *p* < 0.001) [20]. Regarding compliance, a total of 92% of the patients in the TNT group received all six planned cycles of mFOLFIRINOX. The overall incidence of grade 3–4 adverse events (AEs) in patients in the experimental arm was 46%, comparable to what was observed in the control arm, with neutropenia and diarrhea as the most frequent AEs. Updated results after a median follow-up of 7 years were presented at ASCO 2023. The benefit in DFS was maintained with a median DFS of 67.6% in the TNT arm vs. 62.5% in the control arm. An increase in MFS, cancer-specific survival, and OS was also reported, with a 5-year OS rate of 81.9% vs. 76.1% [21].

The RAPIDO trial investigated a consolidation TNT approach, which consisted of SCRT followed by chemotherapy (six cycles of CAPOX or nine cycles of FOLFOX) and TME, compared to standard LCRT. The study randomized 912 patients with high-risk stage II and III rectal cancer, defined as having at least one of the following criteria at MRI staging: cT4a, cT4b, extramural vascular invasion, cN2 (metastasis in four or more locoregional lymph nodes), involved mesorectal fascia, or enlarged lateral lymph nodes [22]. The primary endpoint was 3-year disease-related treatment failure (DrTF), which included LR, distant metastasis, new primary colon tumor, or treatment-related death, while secondary endpoints were pCR, locoregional failure, OS, and safety [22]. The TNT experimental arm demonstrated an improved DrTF rate compared to the control arm (23.7% vs. 30.4%, HR = 0.75; *p* = 0.019) and reached secondary endpoints with a doubled pCR rate (28.4% vs. 14.3%) and a lower rate of distant metastases (20.0% vs. 26.8%, *p* = 0.005). However, despite the observed increase in pCR, locoregional failures in the experimental group were non-significantly higher than in the standard-of-care group (8.3% vs. 6.0%; *p* = 0.12). Regarding safety, grade 3 or higher adverse events during preoperative treatment occurred in 48% of patients in the experimental group, compared with 25% in the standard-of-care group, where 34% of G3-4 AEs were observed during adjuvant treatment. Despite the positive results, the RAPIDO trial has some limitations that should be considered. Notwithstanding the observed increase in pCR, LRs in the experimental group were numerically higher than in the standard of care group (8.3% vs. 6.0%; *p* = 0.12) in the initial report. A recent update of trial results with longer follow-up [23] showed a significantly increased LR rate in the TNT arm, compared with the standard-of-care group(12% vs. 8% respectively, *p* = 0.007). This finding suggests that, in patients with high-risk features, consolidation chemotherapy cannot compensate for a suboptimal radiotherapy strategy in terms of local control of disease. Another concern is related to the optional adjuvant therapy planned by the study in the control arm and administered only in 47% of resected patients, thus making the control arm not an adequate comparator.

The phase III STELLAR trial evaluated a TNT strategy similar to RAPIDO, with patients randomized to SCRT followed by consolidation chemotherapy with four cycles of XELOX, surgery, and two postoperative cycles of XELOX, versus standard LCRT and adjuvant chemotherapy with six cycles of XELOX [24]. The study demonstrated non-inferiority for the primary endpoint, 3-year DFS, in patients receiving TNT versus standard LCRT (64.5% vs. 62.3%, respectively; *p* < 0.001 for non-inferiority). Interestingly, the three-year OS was superior, at 86.5% (95% CI, 82.1 to 90.8), in the TNT group compared with 75.1% (95% CI, 69.4 to 80.8) in the LCRT group (HR = 0.67, 95% CI, 0.46 to 0.97; log-rank, *p* = 0.033), while no significant difference in MFS or LR rate between the groups was observed. Even though the compliance rate was high in the experimental arm, TNT was associated with a significant increase in the rate of grade 3–4 toxicity (26.5% vs. 12.6%; *p* < 0.001) and with a lower completion rate (82.6% vs. 95.2%) compared to LCRT.

### 2.2. Omission of Radiotherapy

Pelvic radiation has traditionally played a leading role in the neoadjuvant treatment of LARC, either alone or in combination with chemotherapy, and has allowed a significant reduction in the LR rate. However, its short-term and long-term toxic effects, particularly concerning bowel and sexual function, together with improvements in systemic chemotherapy, have recently called its role into question.

In the phase III Chinese FOWARC trial, 495 LARC patients were randomly assigned to neoadjuvant treatment with 5-Fluorouracil plus radiotherapy, mFOLFOX6 plus radiotherapy, or mFOLFOX6 alone [25]. The primary endpoint of the study, three-year DFS, was 72.9%, 77.2%, and 73.5% respectively (*p* = 0.709). No significant difference was observed in terms of LR rate or OS, thus the study failed to demonstrate an improvement in DFS with the use of FOLFOX. Nevertheless, the lack of a significant difference in outcomes between mFOLFOX6 without radiotherapy and 5-Fluorouracil with radiotherapy was intriguing and suggested further investigation of this strategy.

Recently, the phase III non-inferiority PROSPECT trial assessed an alternative strategy of neoadjuvant treatment with FOLFOX and selective use of LCRT reserved for patients whose tumors responded poorly [26]. The study included 1128 patients with rectal cancer staged as T2N+, T3N0, or T3N+ who were randomized to standard preoperative LCRT or experimental neoadjuvant chemotherapy with FOLFOX for six cycles, with LCRT given only if the primary tumor decreased in size by less than 20% or if FOLFOX was discontinued because of side effects. Postoperative chemotherapy was suggested in both arms. The primary endpoint of the study was DFS, while secondary endpoints included OS, LR, R0 resection, pCR, and toxic effects. After a median follow-up of almost 5 years, neoadjuvant FOLFOX was non-inferior to LCRT for DFS (HR = 0.92; 90.2% confidence interval [CI], 0.74 to 1.14; *p* = 0.005 for non-inferiority). Five-year DFS was 80.8% (95% CI, 77.9 to 83.7) in the FOLFOX group and 78.6% (95% CI, 75.4 to 81.8) in the LCRT group. No significant differences were found in terms of OS (HR = 1.04; 95% CI, 0.74 to 1.44) and LR (HR = 1.18; 95% CI, 0.44 to 3.16), with a low rate of LR in both arms (1.8% vs. 1.6%) because of eligibility criteria. Among the 585 patients enrolled in the FOLFOX arm, only 53 (9%) received LCRT. The pCR rate was 21.9% in the FOLFOX group and 24.3% in the LCRT group. Regarding safety, a higher incidence of G3-4 adverse events was reported in the FOLFOX group compared to the LCRT group during the neoadjuvant phase (41.0% vs. 22.8%), while this pattern reversed in the adjuvant part of the study. A patient-reported outcome analysis showed that at 12 months after surgery, patients assigned to FOLFOX reported significantly lower rates of fatigue and neuropathy and better sexual function compared to LCRT [27].

These findings suggest that the omission of radiotherapy may represent a safe and viable option in a subset of patients with LARC, without high-risk features, who are willing to avoid some long-term toxicities associated with radiotherapy, such as young patients aiming for fertility preservation.

### 2.3. Induction vs. Consolidation Chemotherapy

As previously mentioned, two main different TNT strategies have been evaluated in clinical trials, utilizing systemic chemotherapy either before (induction) or after (consolidation) LCRT or SCRT. Both strategies have potential advantages and drawbacks. In particular, induction chemotherapy may allow for the early treatment of micrometastatic disease with the potential risk of hampering the efficacy of subsequent CRT through the selection of resistant tumor clones, while upfront CRT may reduce compliance with consolidation chemotherapy. Currently, the optimal sequence of treatments has not been established, but a few clinical studies have compared these two approaches, offering some interesting insights.

In the CAO/ARO/AIO-12 phase II study, 306 patients with stage II–III rectal cancer were enrolled and randomized to receive oxaliplatin-based chemotherapy (three cycles of FOLFOX) either before (induction, group A) or after (consolidation, group B) fluorouracil-oxaliplatin-based LCRT. The primary endpoint of the study was pCR, and the trial utilized a pick-the-winner design based on the hypothesis of an increased pCR of 25% after TNT compared to the standard 15% after preoperative chemoradiotherapy [28]. The study showed a pCR rate of 17% for group A and 25% for group B, indicating that only consolidation chemotherapy met the predefined statistical hypothesis. Notably, following the design of the study, the median interval between completion of chemoradiotherapy and surgery was doubled in group B compared to group A (90 vs. 45 days), which may have significantly contributed to the higher observed pCR rate. Compliance with chemoradiotherapy was better in the consolidation arm, with 97% of patients able to receive full-dose radiotherapy and 87% receiving full-dose 5-fluorouracil, compared to 91% and 78%, respectively, in the induction arm. On the other hand, 92% completed all induction chemotherapy cycles in group A compared to 85% in group B. The long-term results of the study have been published recently, showing no difference in DFS between the two treatment arms, with a 3-year DFS of 73% in both groups (hazard ratio, 0.95; 95% CI, 0.63–1.45, *p* = 0.82). The incidence of LR (6% vs. 5%, *p* = 0.67) and distant metastases (18% vs. 16%, *p* = 0.52) were also not significantly different, as well as other secondary endpoints, including toxicity, QoL, or stool incontinence [29]. 

The OPRA trial, a phase II randomized trial, enrolled 324 patients with LARC candidates for abdominoperineal resection or coloanal anastomosis at baseline [30]. Patients were randomly assigned to receive LCRT with either induction or consolidation chemotherapy. The chemotherapy consisted of eight cycles of FOLFOX or six cycles of CAPOX. Tumor restaging was planned within eight (±4) weeks after TNT. Patients who achieved a complete or near-complete response after finishing treatment were offered watch-and-wait (WW), while TME was recommended for those who achieved an incomplete response. The primary endpoint was DFS, while the secondary endpoint was TME-free survival. The 3-year DFS was not different between the two groups (76%) and was similar to the historical comparison (75%). The proportion of patients who preserved the rectum at 3 years in the intention-to-treat population was 53% for the consolidation group and 41% for the induction group (*p* = 0.01), while the proportion of patients who actually preserved the rectum (TME-free survival) was 60% in the consolidation group and 47% in the induction group (*p* = 0.02). At longer follow-up, the 5-year DFS rates were not significantly different between the two arms, with 71% (95% CI, 64 to 79) and 69% (95% CI, 62 to 77) for induction and consolidation treatment, respectively (*p* = 0.68), and the 5-year OS was 88% in both arms. The difference in TME-free survival was maintained. Of the 225 patients initially offered the WW protocol, 42/105 (40%) in the induction group and 33/120 (27%) in the consolidation group developed tumor regrowth during follow-up and were recommended for TME.

**Table 1:** Clinical trials evaluating total neoadjuvant treatment (TNT).

Phase III Clinical Trials Evaluating TNT									
Ref	Study Title	Study	N	Stage	Induction	Radiotherapy	Consolidation	pCR	Rec
Conroy T. [21]	PRODIGE-23	Prospective phase III	461	cT3-4 or N+	FOLFIRINOX	LCRT	None	27.8%	DFS 76% DM 17%
Bahadoer, R.R. [22]	RAPIDO	Prospective phase III	912	II–III *	None	SCRT	CAPOX/FOLFOX	28.4%	DrTF 23.7% LLR 8.3% DM 20%
Jin J. [24]	STELLAR	Prospective phase III	599	cT3-4 or N+	None	SCRT	CAPOX	cCR 11.1%	DFS 64.5% LLR 8.4% DM 22.8%
Omission of radiotherapy									
Ref	Study title	Study		Stage	Chemiotherapy	Radiotherapy	-	pCR	Rec
Deng Y. [25]	FOWARC	Prospective phase III	495	cT3-4 or N+	mFOLFOX6	None	-	6.5%	DFS 73.5% LR 1.8%
Schrag D. [26]	PROSPECT	Prospective phase III	1128	cT2-3 or N+	FOLFOX	CTRT in selected cases **	-	21.9%	DFS 80.8% LR 1.8%
Induction vs. consolidation chemoterapy									
Ref	Study title	Study		Stage	Induction	Radiotherapy	Consolidation	pCR	Rec
Fokas E. [28]	CAO/ARO/AIO-12	Prospective phase II	306	II–III	FOLFOX (A)	LCRT	FOLFOX (B)	17% (A) 25% (B)	LLR 6% vs. 5% DM 18% vs. 16%
Garcia-Aguilar J. [30]	OPRA	Prospective phase II	324	II–III	FOLFOX (A)	LCRT	FOLFOX (B)	10% (A) 8% (B)	5y DFS 71% vs. 69%

* High-risk defined as having at least one of the following criteria at MRI staging: cT4a, cT4b, extramural vascular invasion (EMVI), cN2 (metastasis in four or more locoregional lymph nodes), involved mesorectal fascia (MRF), or enlarged lateral lymph nodes. ** Primary tumor decreased in size by less than 20% or FOLFOX discontinued because of side effects.

## 3. Immune Checkpoint Inhibitors (ICIs)

ICIs are a class of immunotherapy agents designed to target specific regulatory proteins involved in modulating immune responses, such as programmed death 1, programmed death-ligand 1, and cytotoxic T-lymphocyte-associated protein 4. By blocking these checkpoints, these inhibitors unleash the immune system’s ability to recognize and attack cancer cells, thereby enhancing anti-tumor immune responses [18]. Trials investigating the efficacy of ICIs in the treatment of LARC are summarized in Table 2.

### 3.1. Mismatch Repair-Deficient (dMMR) or Microsatellite Instability-High (MSI-H) LARC

The landscape of neoadjuvant therapy for LARC has been significantly impacted by the exploration of ICIs, particularly in the context of dMMR or MSI-H tumors (Table 2) These tumors, which account for approximately 3% of rectal cancer cases, arise from deficient mismatch repair systems, resulting in the accumulation of mutations within microsatellite DNA regions. Patients with MSI-H/dMMR LARC have distinct clinicopathological and molecular features, including higher tumor-infiltrating lymphocytes and overexpression of immune checkpoint receptors like programmed cell death protein 1 (PD-1) and programmed death-ligand 1 (PD-L1), making them particularly sensitive to immunotherapy.

A seminal study conducted by Cercek et al. investigated the application of PD-1 blockade in stage II and III rectal cancer with MMR deficiency [31]. In this prospective phase II trial, the objective was to evaluate the effectiveness of dostarlimab, an anti-PD-1 monoclonal antibody, in patients diagnosed with dMMR LARC. The primary goal was to ascertain the sustained complete clinical response (cCR) or pathological complete response (pCR) subsequent to dostarlimab treatment, administered with or without chemoradiotherapy. The study yielded remarkable findings, with all enrolled patients achieving a cCR rate following neoadjuvant PD-1 blockade. Consequently, no patients required subsequent chemoradiotherapy or underwent surgical intervention, and no instances of disease progression or recurrence were observed. In January 2023, the US Food and Drug Administration approved dostarlimab for the treatment of dMMR/MSI-H LARC.

Chen et al. recently published the results of an open-label, single-center phase 2 trial aimed at assessing the efficacy of neoadjuvant PD-1 blockade using sintilimab in patients with dMMR LARC [32]. The primary objective was to evaluate the response rate induced by neoadjuvant treatment with sintilimab. Not all patients underwent surgery after the neoadjuvant treatment. Among 16 evaluable patients, 12 (75%) achieved a (pathological or clinical) complete response following treatment. The study thus reinforces the potential of PD-1 blockade with sintilimab as an effective neoadjuvant treatment strategy for dMMR LARC.

Another study by Zhang et al. provided valuable insights into the real-world effectiveness and safety profile of neoadjuvant immunotherapy with a single agent PD-1 inhibitor in dMMR/MSI-H colon and rectal cancer, contributing to the growing body of evidence supporting the use of immunotherapy in this context [33]. Among the 32 enrolled patients, 3 with LARC achieving complete clinical response (cCR) adopted the watch-and-wait (W&W) strategy, while for the remaining 29 patients who underwent surgery, the rates of PR and pCR were 100% and 75.9%, respectively.

The multicenter cohort study by Yang et al. investigated the management of dMMR or MSI-H LARC following neoadjuvant anti-PD-1 monotherapy, exploring the possibility of avoiding surgery in the case of cCR [34]. In fact, 7 of 20 enrolled patients with cCR adopted a W&W strategy. The study showed a cCR rate of 90% and no cases of local or distant recurrence. The findings suggest that patients with dMMR/MSI-H LARC may be considered for excusal from surgery after receiving neoadjuvant anti-PD-1 monotherapy, highlighting the importance of personalized treatment strategies in LARC management.

Moreover, two small and retrospective studies further underscored the efficacy and safety of PD-1 inhibitors, providing additional evidence supporting the integration of immunotherapy into the neoadjuvant setting for dMMR LARC [35,36]. These investigations collectively highlight the evolving role of ICIs in reshaping treatment paradigms, with potential implications for surgical decision-making in specific patient cohorts.

### 3.2. Mismatch Repair-Proficient (pMMR) or Microsatellite Stability (MSS) LARC

Several studies have explored the activity and safety of ICIs in patients with pMMR/MSS LARC. In the NICHE trial, which investigated the combination of Nivolumab and Ipilimumab, fifteen of the enrolled patients were pMMR, and four of them (27%) achieved a pCR [37]. Despite the encouraging results achieved in the above-mentioned study, the benefits from immunotherapy are commonly considered to be restricted to MSI tumors [38]. MSS is thus recognized as the main resistance factor to ICIs and, given its prevalence, is a challenging drawback to overcome. Efforts have been made on two fronts to address this limitation: on a translational research front, the identification of ICI-sensitive MSS subsets has been pursued, while on the clinical research front, combination strategies have been proposed to sensitize MSS tumors to immunotherapy. 

Regarding the identification of molecular ICI-sensitive MSS subsets, some potential molecular markers have been identified. Tumor mutational burden (TMB) has gained interest in this field. It is widely recognized that dMMR/MSI-H patients are characterized by high mutational rates. Nonetheless, a small proportion of non-MSI-H patients could harbor TMB-high status. One cohort published by Fabrizio et al. demonstrated that 2.9% of MSS CRC patients harbor TMB-H status [39]. POLE/POLD1 has also gained interest in this context by enabling the identification of non-MSI-H patients, possibly sensitive to immunotherapy [40]. Although promising, these markers are derived from studies performed on patients with metastatic disease. Moreover, non-POLE, non-TMB-H tumors are supposed to still represent the majority of MSS patients. 

Regarding combination strategies, several treatments have been investigated to enhance ICI sensitivity (Table 2). Associations with tyrosine kinase inhibitors (TKI) and anti-VEGF have already been explored in the metastatic setting. In the localized setting, the sensitizing role of radiotherapy has gained interest [41], and several studies have been conducted on this topic. In the VOLTAGE-A trial, patients with LARC were enrolled if a cT3 or cT4 stage was present at diagnosis, regardless of the nodal involvement. Two cohorts were created in this study, with MSS and MSI patients, respectively. In the MSS cohort, 37 patients were included, and a 30% pCR was achieved [42]. Similarly, the PANDORA trial investigated the efficacy of Durvalumab as a consolidation treatment after LCRT. Patients with local nodal involvement or with cT3/cT4 LARC could be included. pCR was achieved in 34.5% of cases. Notably, 46 out of the 48 patients with available microsatellite status were MSS [43]. Lin et al. investigated the activity of the anti-PD-1 Camrelizumab associated with CAPOX after SCRT in patients affected by LARC, regardless of the MS status. A total of 26 of the included patients were pMMR [44]. A 46% pCR was achieved. The role of the anti-PD-L1 Avelumab has also been investigated. The AVERECTAL trial explored the activity of this molecule when administered in combination with FOLFOX after SCRT. A promising 37% pCR was detected in the MSS cohort [45]. The AVANA trial is an Italian phase II study that has investigated the preoperative efficacy of six cycles of Avelumab (10 mg/kg) administered every two weeks after standard LCRT. The presence of at least one of these criteria was required for inclusion: cN+, cT4, and high-risk cT3. A total of 23% of the 96 evaluable patients showed a pCR. Microsatellite status was available for only 39 patients, and, surprisingly, all of them were MSS except for one [46]. 

Novel potential agents are on the way. Some interesting signals of efficacy have been detected in patients treated with the combination of Botensilimab, a novel anti-CTLA-4 antibody, and Balstilimab, a next-generation PD-1 inhibitor [47]. This promising therapeutic strategy has already provided interesting results in the metastatic setting [48] and is currently under investigation in patients with LARC.

## 4. Non Operative Management (NOM)

Neoadjuvant LCRT followed by radical surgery represents the standard approach for treating LARC [4]. Following LCRT, approximately 15–27% of patients achieve a pCR, defined as the absence of viable tumor cells in the pathological specimen [49]. This rate appears to be even higher in patients undergoing TNT [21,22]. A recent meta-analysis encompassing 3579 patients from 15 trials demonstrated that TNT was associated with a pCR rate of 22.7% compared to 13.6% in the standard treatment group, with a pooled odds ratio of 1.85 (*p* < 0.0001) [50]. Furthermore, the pCR rate in MSI/dMMR rectal cancer treated with ICIs may be even higher, showing promising outcomes in the most recent data [31,32]. These findings, combined with the significant mortality and short- and long-term morbidity associated with surgery (including bowel, urinary, and sexual dysfunction due to nerve and sphincter damage), have sparked a growing interest in NOM for patients who achieve a cCR after neoadjuvant CTRT. cCR is defined as the absence of residual tumor signs upon reassessment, which typically includes digital rectal examination, endoscopy, and radiological imaging.

The concept of NOM was first introduced in 2004 [51]. In this study, the nonoperative group exhibited 5-year OS and DFS rates of 100% and 92%, respectively, compared to 88% and 83% in the surgically resected group. Despite initial skepticism, the “watch-and-wait (WW) strategy” has gained traction in recent years, leading to a notable increase in the proportion of patients and surgeons opting for organ preservation approaches in rectal cancer [52]. Table 3 summarizes the findings of studies comprising more than 40 cases in the WW group.

The major concern regarding the NOM strategy is the risk of LR, which appears to be higher compared to patients undergoing radical surgery [53]. An analysis from 2018, comprising data from 880 patients in the International Watch and Wait Database, reported an LR rate of 25% within the initial 2 years of follow-up [12]. However, a 2023 analysis comparing patients undergoing TME or WW post-TNT demonstrated a lower LR rate (9.9%) [13]. The majority (83–94%) of local regrowths were observed within 2 years in the conservative group [54,55,56]. The likelihood of remaining free from LR increased over time, with patients maintaining sustained cCR for 3 years having an approximately 5% risk of recurrence [57]. LR predominantly occurs in the bowel wall (97% of cases), allowing for salvage surgery in most patients, with reported rates of R0 resection reaching 97.5% in some studies [54,58,59]. Long-term results from the OPRA trial indicated similar rates of R0 resection in patients undergoing radical surgery post-regrowth and those undergoing radical surgery post-neoadjuvant therapy (91% vs. 90%, *p* = 1.0) [56]. Sphincter-saving surgery is feasible in nearly half of patients requiring surgery after LR, comparable to those undergoing radical surgery after LCRT [56]. However, a small Spanish study conducted in 2023 found a higher rate of permanent stomas in salvage surgery after NOM compared to patients undergoing radical surgery (48.5% vs. 20%, *p* < 0.01) [60].

The incidence of distant metastases appears comparable between patients undergoing radical resection and those opting for the NOM strategy [61]. A meta-analysis from 2021 reported distant metastases rates of 10.21% vs. 8.66% (*p* = 0.67, OR 1.12) for the WW and radical surgery groups, respectively [62]. However, local regrowth appears to be a risk factor for distant metastasis development, with patients experiencing LR showing a 5-fold higher risk compared to those without LR [63,64,65]. The heightened risk may arise from a more aggressive biological phenotype, contributing to both LR and the development of metastatic disease. Alternatively, some suggest that metastases may arise from the uncontrolled growth of the primary tumor [66].

Both DFS and OS seem comparable between patients undergoing salvage surgery after NOM and those opting for radical surgery post-neoadjuvant LCRT [58,61,67,68,69]. However, a 2015 Brazilian study reported inferior DFS in patients undergoing WW compared to those undergoing radical surgery (60.9% vs. 82.8%, *p* = 0.011) [70]. Notably, patients undergoing WW often present with more distal tumors, a characteristic associated with lower survival rates (*p* = 0.011). When focusing solely on low rectal tumors in both groups, no significant differences in OS (85.8% vs. 71.7%, *p* = 0.970) were observed, and the discrepancy in DFS was not statistically significant (*p* = 0.081).

The NOM approach could serve as a viable strategy to avoid surgical trauma in patients with cCR; however, careful patient selection and comprehensive information dissemination are crucial. Presently, there exists no consensus on the follow-up protocol for patients considered for the WW approach. The NCCN panel recommends a monitoring regimen comprising digital rectal examination and proctoscopy every 3–4 months for the initial 2 years, followed by semiannual evaluations for 3 years, alongside pelvic MRI every 6 months for at least 3 years [71].

**Table 3:** Clinical trials evaluating the WW strategy.

First Autor	Year	Study Design	Control Group	Number of Patients		DFS (WW vs. Control)	
				Control Group	WW Group	3-Years	5-Years
Araujo [70]	2015	Retrospective cohort study	TME	69	42		60.9% vs. 82.8% (*p* 0.011)
Martens [72]	2016	Prospective cohort study	TEM	15	85	[LRFS] 85.8% vs. 80% (*p* 0.57)	
Smith [64]	2019	Retrospective case series analysis	TME	136	113		75% vs. 92% (*p* N.D.)
Beard [65]	2020	Retrospective cohort study	RS	42	53	[LRFS] 85 vs. 92% (*p* 0.36)	
Wang [69]	2021	Multicenter retrospective cohort study	RS	94	94	[LRFS] 98% vs. 98% (*p* 0.506)	
Najami [73]	2021	Observation descriptive cohort study	LE	22	42	74.9% vs. 66.2% (*p* N.D.)	
Han [61]	2022	Prospective cohort study	TME	26	58	81.1% vs. 84.6% (*p* 0.819)	
Wang [55]	2023	Retrospective cohort study	RS	171	89		93.3% vs. 92% (*p* 0.66)

DFS: disease-free survival; LE: local excision; LRFS: local recurrence-free survival; RS: radical surgery; TEM: trans-anal endoscopic microsurgery; TME: total mesorectal excision.

## 5. Circulating Tumor DNA (ctDNA) Evaluation in LARC Patients

To improve the treatment management of LARC patients, trustworthy biomarkers of treatment efficacy are needed. In this context, the evaluation of ctDNA should help identify patients who would benefit from WW policies and identify those who may need to have their systemic treatment intensified due to a high risk of metastasis.

ctDNA is released into the bloodstream by living cancer cells during cell death, constituting a minute fraction of cell-free DNA. It can be detected by either tumor-informed assays, where probes are designed according to mutations identified in a patient’s tumor tissue, or by tumor-agnostic assays, which are independent of prior tumor genomic knowledge of the patient. The former shows high analytical sensitivity, the latter may offer a more rapid turnaround time with reduced cost [74].

Available data on ctDNA in LARC patients encompass its utility as a marker of both minimal residual disease (MRD) post-definitive local treatment and treatment response to neoadjuvant therapy [75,76,77,78,79,80]. Regarding MRD, consistent findings indicate that patients with detectable postoperative ctDNA face a higher recurrence risk compared to those with undetectable ctDNA [81,82,83,84]. Thus, assessing MRD status enables risk stratification and facilitates determining patients likely to benefit from adjuvant chemotherapy.

The relationship between preoperative ctDNA and surgical outcomes has been less explored, with recent interest emerging in the additive value of ctDNA levels for monitoring response in neoadjuvant settings [75,76,77,78,79,80]. Several studies confirmed the association between ctDNA and prognosis, but failed to find a significant association between ctDNA status and pCR [84,85,86,87]. No association was found between ctDNA status and pCR in GEMCAD 1402, a phase II randomized, multicenter clinical trial [75]. However, preoperative ctDNA status significantly correlates with postoperative pathological results, indicating its potential as a real-time monitoring indicator reflecting tumor burden [76]. Additionally, undetectable preoperative ctDNA is associated with favorable surgical outcomes, as evidenced by margin-negative, node-negative resections, and neoadjuvant rectal score [85]. 

Tumor-informed ctDNA detection using ultradeep sequencing in LARC patients may offer clinical value for predicting response following neoadjuvant therapy and surgery [86]. Our study, utilizing a personalized tumor-informed ctDNA assay (Signatera™), demonstrated worse DFS in ctDNA-positive patients post-neoadjuvant therapy and surgery compared to ctDNA-negative counterparts [88]. Furthermore, patients remaining ctDNA-positive post-neoadjuvant therapy, irrespective of pCR status, exhibited inferior DFS compared to ctDNA-negative patients [88]. Combining post-neoadjuvant therapy ctDNA status with neoadjuvant rectal score predicted neoadjuvant therapy response, as already shown in previous research [85]. Notably, the sensitivity of ctDNA assays, whether tumor-agnostic or tumor-informed, impacts baseline ctDNA positivity detection, thereby influencing the number of patients for whom ctDNA dynamic monitoring could be useful [75,84,88].

## 6. Discussion

The standard management approach for LARC involves multimodal therapy, integrating radiation, chemotherapy, and surgery. While LCRT and SCRT yield comparable survival outcomes, the latter is associated with reduced rates of pCR and increased LR. Despite the introduction of LCRT, distant metastases remain unaffected. Poor tolerance to adjuvant chemotherapy has driven the exploration of diverse TNT strategies, administering both radiotherapy and chemotherapy before surgery. Recently, three phase III trials, PRODIGE-23, RAPIDO, and STELLAR, compared different TNT strategies to standard LCRT. The PRODIGE-23 study confirmed the tolerability and efficacy of the triplet regimen mFOLFIRINOX as induction chemotherapy in a TNT strategy [20,21]. However, despite the favorable safety profile of the treatment compared to the metastatic setting, this intensive regimen should be reserved for selected patients. Moreover, due to the lack of an arm with a neoadjuvant doublet regimen such as FOLFOX/XELOX, it is difficult to assess the added benefit of irinotecan. Additionally, it should be noted that the trial included mandatory adjuvant treatment in the experimental arm, which was started in about 70% of patients, confirming the poor compliance with post-operative chemotherapy in this setting. RAPIDO and STELLAR trials investigated a consolidation TNT approach. While the RAPIDO trial yielded promising outcomes, it carries certain limitations worth noting [22,23]. Despite the observed increase in pCR, the experimental group exhibited numerically higher rates of LR compared to the standard-of-care group. This suggests that consolidation chemotherapy may not fully compensate for suboptimal radiotherapy strategies in patients with high-risk disease features. Additionally, concerns arise regarding the optional adjuvant therapy in the control arm, which was administered to only 47% of resected patients, rendering the control arm an inadequate comparator. The phase III STELLAR trial, assessing a TNT strategy akin to RAPIDO, found no significant differences in LR between TNT and LCRT [24]. The differences in clinical outcome observed between STELLAR and RAPIDO trials may partly be related to different eligibility criteria, with more patients with cT4 and cN2 disease in RAPIDO compared to STELLAR, but also in trial design. Indeed, in the STELLAR trial, an equivalent number of chemotherapy cycles were planned in the control and experimental arms, and this may partly explain the absence of a difference in DFS. Overall, although non-inferiority of the experimental arm was shown in the study, some important confounding factors, such as increased toxicity, lack of a benefit in endpoints related to disease control (DFS, MFS, and LR), and the significant proportion of patients not undergoing surgery (about 20%) suggest a cautious interpretation of trial results. 

Currently, the optimal treatment sequence remains undefined, but some clinical trials have compared induction and consolidation chemotherapy regimens, providing valuable insights. Considering the findings from the CAO/ARO/AIO-12 and OPRA trials together, the use of induction chemotherapy results in better chemotherapy compliance but lower radiotherapy compliance compared to consolidation treatment [28,29]. No differences in terms of DFS or OS have been observed in the two trials between these strategies, but the use of consolidation chemotherapy seems to be associated with higher rates of pCR and clinical response rate, leading to improved TME-free survival. These results may be partly explained by the longer time interval between completion of LCRT and surgery, which may allow for a deeper effect of radiation therapy. Indeed, in the OPRA trial, the median interval from the end of chemoradiotherapy to restaging was 8.0 weeks in the induction arm and 28 weeks in the consolidation arm, and similarly, in the CAO/ARO/AIO-12 trial, the interval after chemoradiotherapy was doubled in the consolidation arm. In both trials, the increase in the time interval between radiotherapy and surgery did not increase surgical morbidity.

Ongoing trials are investigating optimal treatment sequences, evaluating long-term outcomes, and exploring novel therapeutic approaches (Table 4). There is probably no optimal TNT sequence for all LARC patients, but the treatment strategy should be individualized based on the patient’s characteristics and treatment goals. For example, consolidation chemotherapy after chemoradiotherapy may represent the optimal choice in large cT4 or mesorectal fascia-involving tumors or when the organ preservation strategy is pursued. On the other hand, the use of induction chemotherapy, considering the better compliance to chemotherapy, may be preferable in patients with a high risk of micrometastatic disease, such as those with EMVI or N2 tumors.

Radiation therapy continues to play a pivotal role in the multimodal treatment of LARC, and ongoing advancements in radiation techniques can significantly enhance the success of neoadjuvant therapy. In particular, intensity-modulated radiation therapy (IMRT) and image-guided radiation therapy (IGRT) have significantly improved treatment precision and outcomes [89]. IMRT allows for the delivery of highly conformal radiation doses, minimizing exposure of surrounding healthy tissues and reducing toxicities. Combined with IGRT, which provides real-time imaging guidance during treatment delivery, clinicians can accurately target tumors while adapting treatment plans based on anatomical changes, ensuring optimal tumor coverage and sparing of critical structures. Future developments in this field are anticipated to focus on further refining IMRT and IGRT techniques, integrating advanced imaging modalities such as MRI and PET-CT for improved tumor visualization and delineation, and exploring novel strategies such as proton therapy to enhance treatment efficacy and minimize side effects. These advancements hold promise for enhancing the therapeutic outcomes and quality of life for patients with LARC undergoing neoadjuvant treatment. On the other hand, recent clinical trials are currently exploring the feasibility of omitting radiation therapy in the neoadjuvant treatment of LARC. This approach is based on the hypothesis that chemotherapy (or even immunotherapy) alone may achieve comparable outcomes while minimizing the risk of radiation-related toxicities.

The presence of MSI-H/dMMR in rectal cancer can serve as both a negative predictor for the efficacy of standard neoadjuvant chemoradiotherapy and a positive predictor for the effectiveness of pre-operative immunotherapy. Studies, such as the phase II trial conducted by Cercek et al., have demonstrated the efficacy of anti-PD-1 monoclonal antibodies, such as dostarlimab, in inducing sustained clinical cCR in patients with MSI-H/dMMR LARC [31]. Future prospects in this field are promising, with several ongoing clinical trials aiming to further elucidate both efficacy and safety profiles (Table 5).

These studies may provide valuable insights into optimizing treatment strategies, including the selection of appropriate immunotherapeutic agents, treatment combinations, dosing regimens, and patient selection criteria. Additionally, these trials may shed light on the potential role of immunotherapy in enhancing pathological responses, improving long-term outcomes, and providing the opportunity to avoid chemoradiotherapy and surgery in selected cases of MSI-H LARC. While these findings are based on limited studies, they suggest a promising short-term efficacy of preoperative immunotherapy as a single treatment modality in patients with LARC. Several studies have also explored ICIs in patients with pMMR/MSS LARC. While the benefits of immunotherapy are mostly observed in MSI tumors, efforts are underway to identify MSS subsets sensitive to ICIs and explore combination strategies to enhance their efficacy (Table 5). Moreover, ongoing trials are investigating novel agents like Botensilimab and Balstilimab in LARC patients.

Increasing interest in NOM has emerged due to significant surgical morbidity and mortality, prompting a notable rise in organ preservation approaches. Concerns regarding the risk of LR persist, although rates have improved over time, with most local regrowth occurring within the initial 2 years. The incidence of distant metastases appears comparable between radical resection and NOM, yet careful patient selection and comprehensive information dissemination are crucial given differences in outcomes.

Reliable biomarkers like circulating tumor DNA (ctDNA) are crucial for optimizing treatment in LARC patients, aiding in identifying candidates for WW and those requiring intensified therapy. Utilizing tumor-informed ctDNA assays may provide real-time monitoring of tumor burden, guiding treatment decisions and improving outcomes in neoadjuvant settings. Despite its increasing use in clinical studies, ctDNA analysis remains underutilized in routine care for LARC patients due to study heterogeneity regarding ctDNA assay selection, timing of ctDNA collection, treatment variations, and follow-up duration. Incorporating ctDNA analysis into larger randomized trials is imperative to evaluate ctDNA dynamics across neoadjuvant treatment phases and surveillance, thus informing its potential clinical utility, particularly in the context of TNT and organ preservation approaches.

## 7. Conclusions

The multimodal treatment strategy for LARC is rapidly evolving, representing a significant departure from previous static phases. Our work entails a critical analysis of recent clinical trials on this topic, alongside insights into ongoing trials poised to substantially influence near-future clinical practice. Neoadjuvant therapy remains paramount, with TNT recommended for all patients presenting high-risk features. Additionally, ICIs are indicated for all patients with dMMR/MSI-H tumors. Furthermore, the enrollment of patients in WW protocols and ctDNA studies is highly recommended whenever feasible, offering valuable insights into treatment efficacy and patient outcomes. Optimal outcomes in LARC hinge upon individualized treatment decisions made collaboratively within a multidisciplinary team, considering patient characteristics and treatment objectives.

## Figures and Tables

**Table 2:** Trials investigating the efficacy of ICIs in the treatment of LARC.

ICI	Study Title/ Reference	Patients (N°)	Induction	Radio Therapy	Consolidation	pCR
dMMR/MSI						
Dostarlimab	NCT04165772	12	Dostarlimab	LCRT	None	100%
Sintilimab	NCT04304209	17	Sintilimab	None	None	75%
Tislelizumab, Sintilimab, Pembrolizumab	Zhang X.	32	None	Not Specified *	None	100%
Tislelizumab, Sintilimab, Pembrolizumab	Yang R.	20	None	None	None	90%
Pembrolizumab, Nivolumab +/− CT	Kothari A.	9	None	None	None	89%
Pembrolizumab, Nivolumab +/− CT **	Demisse R.	3	None	Not Specified ***	None	100%
MSS						
Nivolumab	VOLTAGE-A	37	Nivolumab	LCRT	None	30% [7]
Camrelizumab	NCT04231552	26	None	SCRT	CAPOX + Camrelizumab (2 × 21 days cycles)	46.2% [9]
Durvalumab	PANDORA	46	None	LCRT	Durvalumab	34.5% [8]
Avelumab	AVERECTAL	26	None	SCRT	FOLFOX + Avelumab	37% [10]
Avelumab	AVANA	38	None	LCRT	Avelumab	23% [11]

* Two patients underwent neoadjuvant chemoradiotherapy. ** One patient received neoadjuvant Pembrolizumab and FOLFOX. *** One patient received neoadjuvant chemoradiotherapy, then ICI at progression.

**Table 4:** Ongoing clinical trials evaluating total neoadjuvant treatment (TNT).

NCT Number	Treatment Strategy	Status	Phase
NCT03038256	Ctr: CTRT → S → ADT Exp: CTRT → CAPOX x4 → S Exp1: CTRT → CAPOX x6 → S	Recruiting	Phase II
NCT05054959	Exp1: CTRT → CAPOX x6 → S Exp 2: CAPOX x4 → CTRT → CAPOX x2 → S	Recruiting	Phase II
NCT05673772	Exp1: SCRT → FOLFOX x4 → S → ADT Ctr: CTRT → S → ADT	Recruiting	Phase II
NCT05610163	Exp1: CTRT → FOLFOX/CAPOX → S or NOM Exp2: CTRT → FOLFIRINOX → S or NOM Ctr: CTRT → S → ADT	Recruiting	Phase II
NCT04246684	Exp: CTRT (+Oxa) → CT (FOLFOX x6/CAPOX x4) Ctr: SCRT → CT (FOLFOX x9/CAPOX x6) If cCR → NOM If not cCR → S	Recruiting	Phase III
NCT04215731	Exp1: FOLFOXIRI + bevacizumab x4 → FOLFOXIRI x2 → immediate S or CTRT → S according to ycT Exp2: FOLFOX x4 → CTRT → S	Recruiting	Phase III
NCT05646511	Exp 1: SCRT → CAPOX x6 → S or NOM Exp 2: SCRT → CAPOXIRI x6 → S or NOM	Recruiting	Phase III

ADT: adjuvant therapy; ctr: control; cCR: complete clinical response; CTRT: chemo-radiotherapy; exp: experimental; NOM: nonoperative management; S: surgery; oxa: oxaliplatin; SCRT: short-course radiotherapy.

**Table 5:** List of ongoing clinical trials evaluating ICIs in the neoadjuvant setting.

MSI-H Tumors			
NCT Number	Study Title	Study Status	Phases
NCT05645094	Neoadjuvant Envafolimab in Resectable and Locally Advanced MSI-H/dMMR Rectal Cancer	NOT YET RECRUITING	PHASE2
NCT04411524	The Combination of Immunotherapy and Neoadjuvant Chemoradiotherapy in MSI-H Locally Advanced Rectal Cancer	UNKNOWN STATUS	PHASE2
NCT04301557	PD1 Antibody Toripalimab and Chemoradiotherapy for dMMR/MSI-H Locally Advanced Colorectal Cancer	RECRUITING	PHASE2
NCT05723562	A Study of Dostarlimab in Untreated dMMR/MSI-H Locally Advanced Rectal Cancer (AZUR-1)	RECRUITING	PHASE2
NCT04357587	Safety and Feasibility of PD-1 Blockade in the Treatment of dMMR or MSI-H Rectal Cancer	RECRUITING	PHASE2
NCT04304209	Pd1 Antibody Sintilimab ± Chemoradiotherapy for Locally Advanced Rectal Cancer	ACTIVE, NOT RECRUITING	PHASE2
NCT04751370	Testing Nivolumab and Ipilimumab with Short-Course Radiation in Locally Advanced Rectal Cancer	SUSPENDED	PHASE2
NCT05732389	Immunotherapy in Patients with Early dMMR Rectal Cancer (RESET-R)	RECRUITING	PHASE2
NCT04636008	Neoadjuvant treatment of sintilimab plus hypofractionated radiotherapy for MSI-H/dMMR rectal cancer: A prospective, multicenter, phase Ib study	RECRUITING	PHASE2
NCT05645094	Neoadjuvant Envafolimab in Resectable and Locally Advanced MSI-H/dMMR Rectal Cancer	NOT YET RECRUITING	PHASE2
NCT04411524	The Combination of Immunotherapy and Neoadjuvant Chemoradiotherapy in MSI-H Locally Advanced Rectal Cancer	UNKNOWN STATUS	PHASE2
NCT04301557	PD1 Antibody Toripalimab and Chemoradiotherapy for dMMR/MSI-H Locally Advanced Colorectal Cancer	RECRUITING	PHASE2
NCT05723562	A Study of Dostarlimab in Untreated dMMR/MSI-H Locally Advanced Rectal Cancer (AZUR-1)	RECRUITING	PHASE2
NCT04357587	Safety and Feasibility of PD-1 Blockade in the Treatment of dMMR or MSI-H Rectal Cancer	RECRUITING	PHASE2
NCT04304209	Pd1 Antibody Sintilimab ± Chemoradiotherapy for Locally Advanced Rectal Cancer	ACTIVE, NOT RECRUITING	PHASE2
NCT04751370	Testing Nivolumab and Ipilimumab with Short-Course Radiation in Locally Advanced Rectal Cancer	SUSPENDED	PHASE2
NCT05732389	Immunotherapy in Patients with Early dMMR Rectal Cancer (RESET-R)	RECRUITING	PHASE2
NCT04636008	Neoadjuvant treatment of sintilimab plus hypofractionated radiotherapy for MSI-H/dMMR rectal cancer: A prospective, multicenter, phase Ib study	RECRUITING	PHASE2
MSS TUMORS			
NCT05215379	Neoadjuvant Chemoradiation Therapy Combined with Immunotherapy for MSS Ultra-low Rectal Cancer	RECRUITING	PHASE2|PHASE3
NCT04109755	Neo-adjuvant Pembrolizumab and Radiotherapy in Localized MSS Rectal Cancer	RECRUITING	PHASE2
NCT04895137	mFOLFOX6 + Bevacizumab + PD-1 Monoclonal Antibody in Local Advanced MSS CRC	RECRUITING	PHASE2
NCT04833387	PD-1 Antibody Following Preoperative Chemoradiotherapy for Locally Advanced pMMR/MSS Rectal Cancer	ACTIVE, NOT RECRUITING	PHASE2
NCT05731726	Serplulimab Combined with CAPOX + Celecoxib as Neoadjuvant Treatment for Locally Advanced Rectal Cancer	RECRUITING	PHASE2
NCT05972655	Nodes-sparing Short-course Radiation Combined with CAPOX and Tislelizumab for MSS Middle and Low Rectal Cancer	RECRUITING	PHASE2
NCT04940546	Neoadjuvant Safety of Sintilimab + XELOX + Bevacizumab in pMMR/MSS CRLM Patients	ACTIVE, NOT RECRUITING	PHASE1|PHASE2
NCT05858567	Total Neoadjuvant Therapy with Short-course Radiation Followed by Envafolimab Plus CAPOX for MSS Locally Advanced Ultra Low Rectal Adenocarcinoma	RECRUITING	PHASE2
NCT05216653	Preoperative Short-course Radiation Followed by Envafolimab Plus CAPOX for MSS Locally Advanced Rectal Adenocarcinoma (PRECAM)	ACTIVE, NOT RECRUITING	PHASE2
NCT05752136	Preoperative Short-course Radiation Followed by Envafolimab Plus CAPOX for MSS Locally Advanced Rectal Adenocarcinoma	RECRUITING	PHASE3
NCT05815303	XELOX Combined with Cadonilimab Versus XELOX as Neoadjuvant Treatment for Locally Advanced, pMMR Rectal Cancer	RECRUITING	PHASE2
NCT04304209	Pd1 Antibody Sintilimab ± Chemoradiotherapy for Locally Advanced Rectal Cancer	ACTIVE, NOT RECRUITING	PHASE2

## Data Availability

The datasets generated and/or analyzed during the current study are available from the corresponding author upon reasonable request.
